# Transcription Factor FXR Activates DHRS9 to Inhibit the Cell Oxidative Phosphorylation and Suppress Colon Cancer Progression

**DOI:** 10.1155/2022/8275574

**Published:** 2022-10-26

**Authors:** Jinlai Zhao, Yigang Wang, Yang Wang, Jianchao Gao, Haichao Yang, Xiaotang Wu, Hua Li

**Affiliations:** ^1^Gastrointestinal surgery, Tangshan Central Hospital, Tangshan, Hebei 063000, China; ^2^Anus and intestine surgery, Tangshan Central Hospital, Tangshan, 063000 Hebei, China; ^3^Hebei University of Economics and Business, Shijiazhuang, Hebei 050062, China

## Abstract

**Background:**

Colon cancer is a common gastrointestinal malignancy. It has been discovered that Farnesoid X receptor (FXR) plays an imperative regulatory role in multitype cancers in recent years. However, its regulatory mechanism in colon cancer has not been clearly explored. This study intended to explore the molecular regulatory mechanism of FXR and its downstream genes on the malignant progression of colon cancer.

**Methods:**

The mRNA and protein expression of FXR in colon cancer cells were measured by quantitative real-time polymerase chain reaction and Western blot. The effects of FXR on the biological function of colon cancer cells were measured by Cell Counting Kit-8, colony formation, and transwell assays. The downstream target gene of FXR was predicted by bioinformatics analysis and found to be associated with cellular oxidative phosphorylation. The binding relationship between FXR and its downstream gene dehydrogenase/reductase member 9 (DHRS9) was verified through luciferase reporter assay and chromatin immunoprecipitation assay. The changes of oxidative phosphorylation were detected by Western blot and oxygen consumption rate determination. The effect of FXR/DHRS9 axis on the malignant progression of colon cancer cells was further confirmed by rescue experiments.

**Results:**

FXR was underexpressed in colon cancer tissues and cells, and overexpressing FXR could repress the malignant behaviors of colon cancer cells. Besides, DHRS9 was a downstream gene of FXR, and FXR/DHRS9 inhibited the deterioration of colon cancer through inhibiting oxidative phosphorylation. Moreover, promoting FXR expression in colon cancer cells could partially reverse the biological function changes caused by silencing DHRS9 expression.

**Conclusion:**

FXR inhibited the oxidative phosphorylation and inhibited the malignant progression of colon cancer cells via targeting DHRS9.

## 1. Introduction

Colon cancer is a common malignancy. According to the statistical data released by the American Cancer Society, the morbidity and mortality of colon cancer in human is 10.2% and 9.2%, respectively [1, 2]. According to data released by the International Agency for Research on Cancer (IARC) of the World Health Organization, the new cases of colon cancer worldwide in 2020 exceeded 1.14 million, and 580,000 cases died from this disease [3]. As people's lifestyle changes, the incidence rate of colon cancer is rising continuously in developing countries [4, 5]. Although breakthroughs have been made in colon cancer treatment, the prognosis of advanced colon cancer remains unsatisfactory because of distant metastasis and recurrence [6]. The molecular pathogenesis of colon cancer is a continuous multistep process, and understanding the pathogenesis of colon cancer is critical to developing better prognosis and treatment strategies.

Farnesoid X receptor (FXR) is a type of bile acid receptor [7, 8]. Studies have indicated that bile acid is related to the pathogenesis of human malignant tumors, including liver cancer, gastric cancer, and esophageal cancer [9–11]. Human epidemiology and animal studies have demonstrated that the risk of colon cancer is strongly correlated with fecal bile acid concentration [12, 13]. FXR expression level is high in kidney, liver, and adrenal glands but relatively low in fat and heart [14, 15]. FXR binds to DNA (a FXR response element) and participates in the regulation of bile acid and glucose metabolism related genes [16–18]. In addition, accumulating evidence has confirmed that FXR is a key part in human tumorigenesis [19, 20]. Bailey et al. [21] found that FXR mRNA level was reduced in colon polyps, and the reduction is more significantly in colorectal cancer. Also, overexpression of FXR has been shown to restrain the abnormal growth of intestinal cells and the progression of colorectal cancer [22]. Nevertheless, the exact mechanism of FXR in the progression of colon cancer needs to be further elucidated.

As a member of the short chain dehydrogenase/reductase family, dehydrogenase/reductase member 9 (DHRS9) is thought to be associated with in the metabolism of retinol [23]. Soref et al. [23] first characterized the enzyme activity of DHRS9 in airway epithelial cells, and Jette et al. [24] later revealed that DHRS9 mRNA was mainly expressed in the colon, with a low level. Previous studies have confirmed that DHRS9 participated in the biosynthesis of all-transretinoic acid (ATRA) [25]. Since ATRA is a key role in tumorigenesis, it is supposed that DHRS9 has a correlation with tumor occurrence and development [26]. Studies have confirmed the important antitumor activity of DHRS9 and its role in the treatment of various cancers [25, 27–30]. As the lack of retinoic acid biosynthesis is considered to be a mechanism leading to the development of colorectal adenocarcinoma, we hypothesized that there may be a correlation between the dysregulation of DHRS9 expression and the invasiveness of colon cancer. However, the association of DHRS9 expression with malignant progression of colon cancer has not been explored.

In the current study, we determined the expression of FXR in colon cancer and its role in the deterioration of colon cancer, and we further explored its target gene DHRS9 and analyzed DHRS9-associated oxidative phosphorylation mechanism, thus to provide a more sufficient theoretical basis for FXR/DHRS9 regulating the progression of colon cancer.

## 2. Materials and Methods

### 2.1. Bioinformatics Methods

The mRNA expression data (normal: 41, tumor: 480) of colon cancer were collected through The Cancer Genome Atlas (TCGA) (https://portal.gdc.cancer.gov/). The differentially expressed mRNAs (DEmRNAs) were obtained through differential analysis by using the “edgeR” package ,(|logFC| > 2FDR < 0.05). The target transcription factor was identified by literature review. The potential target genes downstream of the target transcription factor was predicted by MotifMap database (http://motifmap.ics.uci.edu) and GTRD database (http://gtrd.biouml.org/#!), and the target gene was determined by the Pearson correlation analysis and literature review. The binding site between the target gene and the target transcription factor was predicted by using JASPAR (http://jaspar.genereg.net/). Gene set enrichment analysis (GSEA) was utilized for the pathway enrichment analysis of target mRNA.

### 2.2. Cell Culture

Human colon epithelial cell line NCM460 (BNCC353657) and colon cancer cell lines, including HCT 116 (BNCC337692), HT-29 (BNCC100164), SW480 (BNCC100604), Lovo (BNCC338601), Caco-2 (BNCC350769), and RKO (BNCC100173) were obtained from BeNa Culture Collection (China). These cell lines were maintained in Dulbecco's Modified Eagle Medium (DMEM) (Gibco, USA) supplemented with 10% fetal bovine serum (FBS) (Thermo Fisher Scientific, USA) at 37°C with 5% CO_2_.

### 2.3. Cell Transfection

The overexpression FXR (oe-FXR), silencing DHRS9 (sh-DHRS9), and their corresponding negative controls (NCs) were procured from GeneChem Company (China). Lipofectamine 2000 (Invitrogen, USA) was employed to transfect the oe-FXR, sh-DHRS9, and their corresponding NCs into HT-29 and SW480 colon cancer cells. Cells were collected 24 h after transfection for following experiments.

### 2.4. Quantitative Real-Time Polymerase Chain Reaction (qRT-PCR)

Total RNA extraction was performed using Trizol reagent (Thermo Fisher Scientific, USA). cDNA was synthesized from the extracted RNA using a High-Capacity cDNA Reverse Transcription Kit (Thermo Fisher Scientific, USA). Real-time qPCR was conducted on QuantStudio 3 PCR instrument (Thermo Fisher Scientific, USA) using SYBR Green fluorescence signal detection kit (Takara, Japan) and corresponding primers (Table 1). The quantitation of the expression level of specific mRNA was performed using 2^−ΔΔCT^.

### 2.5. Western Blot

Radioimmunoprecipitation buffer (Thermo Fisher Scientific, USA) was used to lyse the cells. Cell lysate containing 50 *μ*g of total protein was transferred onto a polyvinylidene fluoride membrane (Millipore, USA) after sodium dodecyl sulphate-polyacrylamide gel electrophoresis (Thermo Fisher Scientific, USA). The membrane and primary antibody were incubated at 4°C overnight. The protein bands were then rinsed with Tris buffer saline plus Tween (TBST) buffer 3 times, 10 min each. Next, the membrane and secondary antibody were incubated at room temperature for 2 h. Chemiluminescence substrate (Thermo Fisher Scientific, USA) was added to observe the protein bands.

The primary antibodies including anti-FXR (ab129089, diluted at 1 : 1000), anti-DHRS9 (ab126074, diluted at 1 : 1000), anti-ATP5D (ab97491, diluted at 1 : 1000), anti-ATP5E (Cat #PA5-104424, diluted at 1 : 1000), anti-NDUFA3 (H00004696-K, diluted at 1 : 1000), and anti-GAPDH (ab9485, diluted at 1 : 2500) were all rabbit-derived antibodies. Anti-FXR, anti-DHRS9, anti-ATP5D, and anti-GAPDH were from Abcam (UK). Anti-ATP5E antibody was from Thermo Scientific (USA). The anti-NDUFA3 antibody was bought from Abnova (China). Goat anti-rabbit IgG H&L (HRP) antibody (Abcam, ab6721, diluted at 1 : 2000, UK) served as the secondary antibody.

### 2.6. Cell Counting Kit-8 (CCK-8) Assay

The transfected HT-29 and SW480 cells (about 3 × 10^3^ cells per well) were plated into the 96-well plate. After 0, 1, 2, and 3 days, 10 *μ*l CCK-8 solution (MedChem Express, USA) was supplemented to each well, and the incubation was continued for 2 h in a 37°C incubator with 5% CO_2_. The OD value was read at 450 nm by a microplate reader (Bio-Rad Laboratories, USA).

### 2.7. Colony Formation Assay

HT-29 or SW480 cells (about 0.4 × 10^3^ cells per well) were planted into the 6-well plates, and the plates were kept in a 37°C incubator with 5% CO_2_ for culture. Fresh medium was replaced every 3-4 d. After 10-14 d, when the spots were visible to the naked eyes, the cells were fixed with 4% paraformaldehyde for 15 min. Later, the cells were treated with 0.1% crystal violet for 20 min. After staining, the excess crystal violet dye in the well was cleaned by using phosphate buffer saline (PBS), and the number of colonies was determined.

### 2.8. Transwell Assay

The HT-29 or SW480 cells (about 1 × 10^5^ cells per well) were first seeded into the upper chamber of the Transwell device with 8 *μ*m aperture with serum-free medium (Corning, USA). Meanwhile, the cell culture medium supplemented with 10% FBS was filled into the lower chamber. After 24 to 48 h of incubation at 37°C, the unmigrated cells in the upper chamber were removed with cotton swabs, while the migrated cells were subjected to fixation (4% paraformaldehyde) and staining (0.1% crystal violet). The image was observed under a microscope (Shanghai Caikon Optical Instrument Co., China), and the relative cell number was calculated. For the determination of cell invasion assay, 50 *μ*l matrix gel was applied to the bottom of the upper chamber before cell inoculation (BD Biosciences, USA), and the rest of the procedure was basically the same as the migration assay.

### 2.9. Dual-Luciferase Reporter Gene Assay

The amplified 3′UTR sequence of DHRS9 mutant (DHRS9-Mut) or wild type (DHRS9-Wt) was imported into the PGL3-basic vector (Addgene, USA) to construct the reporter gene plasmid. The HT-29 cells were then planted into the 96-well plate. Next, the HT-29 cells were cotransfected with oe-FXR/oe-NC and reporter plasmid. The fluorescence intensity in the transfected groups was measured by luciferase activity assay kit (Promega, USA) 48 h after transfection.

### 2.10. Chromatin Immunoprecipitation (ChIP) Assay

ChIP assay was performed using the ChIP kit (CST, #9006, USA). In short, HT-29 cells were crosslinked with formaldehyde for 10 min, and then the crosslinking was terminated by adding 125 nM glycine and reacting for 5 min. The cells were then harvested and treated with ultrasound, till the DNA had an average length of 200-1000 bp. Next, immunoprecipitation was conducted using FXR antibody (Abcam, ab168852, UK) as controls, and the precipitated DNA was amplified using qRT-PCR. The DNA was incubated with cell lysate at 4°C overnight. Then, Dynabeads Protein G (Invitrogen, USA) was added for DNA enrichment for 2 h. IgG (Proteintech, B900620, USA) served as a negative control. Finally, DNA was measured by performing qRT-PCR. The primer sequence 1 of DHRS9 promoter was Forward: 5′-TCCCCTGCTGGTTTGATGATT-3′; Reverse: 5′-AAAATATCCTGCCTTTCCCCCA-3′; Primer sequence 2 was: 5′-AACAGAGTGCATACCCTTTCA-3′; Reverse: 5′-GGCTTATTTTTGTAAAGCAAACTCT-3′.

### 2.11. Clinical Sample Collection

Colon cancer patients (*n* = 15) without any therapeutic treatment were enrolled for sample collection in Tangshan Central Hospital from January 2020 to January 2022. Tumor tissues and the corresponding adjacent tissues were collected form the enrolled colon cancer patients. All the patients signed the informed consent, and the relevant experiments with clinical samples were approved by the ethics committee of Tangshan Central Hospital.

### 2.12. Measurement of Cell Oxygen Consumption Rate (OCR)

OCR was determined using a Seahorse Biosciences XF96 analyzer (North Billerica, USA). The cells were kept in petri dishes for 24 h and then acclimated in XF medium at 37°C for 2 h. OCR measurement was conducted as per the instructions of XF Cell Mito Stress Test Profile. Oligomycin, trifluoromethyl phenylhydrazone (CCCP), and rotenone were successively added, and then the OCR value was determined. The basal oxygen consumption rate = basal oxygen consumption-nonmitochondrial respiration and proton leakage (mean (1)-mean (2)). The maximum oxygen consumption rate = maximum oxygen consumption-nonmitochondrial respiration and proton leakage (max (3)-mean (2)).

### 2.13. Data Analysis

All experiments in this study were independent experiments and were repeated for 3 times. GraphPad Prism 8.0 Software (GraphPad Software, USA) was used for statistics, analysis, and plotting of the data obtained from the experiment. All data in the figures were presented in the form of mean ± standard deviation, and intergroup data comparison was performed using *t*-test or one-way analysis of variance. *P* value was used to judge the significance of difference, asterisk corresponded to the significance level of difference, ^∗^ indicated *P* < 0.05.

## 3. Results

### 3.1. FXR Expression Is Downregulated in Colon Cancer

**Figure 1:**
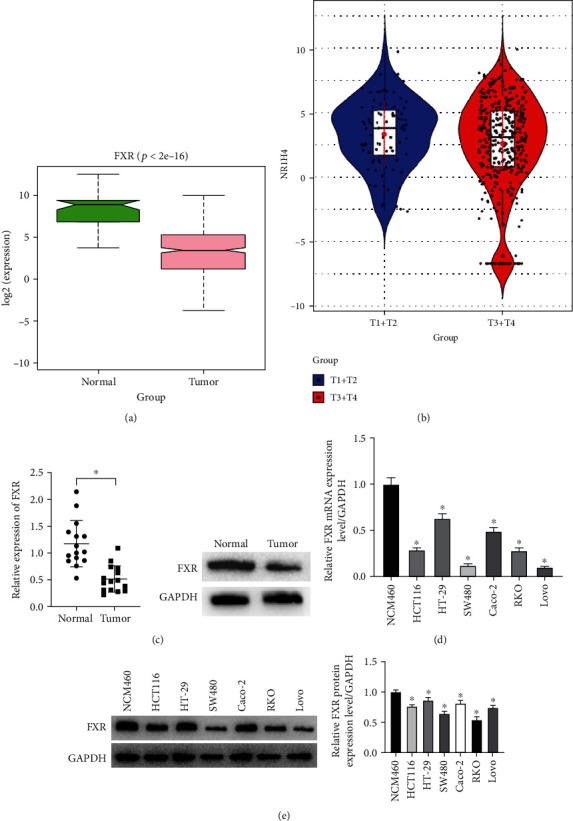
FXR is lowly expressed in colon cancer cell lines. (a). According to TCGA database, the expression of FXR in colon cancer tissues was significantly reduced compared to normal tissues. The green box shows the normal sample and the pink box shows the tumor sample. (b). According to TCGA database, the expression of FXR in T3+T4 colon cancer patients was significantly reduced compared to T1+T2 patients. The blue violin shows the T1+T2 patients, and the red violin shows the T3+T4 patients. (c). qRT-PCR and Western blot were employed to evaluate the mRNA and protein levels of FXR in colon cancer patients. (d and e). qRT-PCR and Western blot were employed to evaluate the mRNA and protein levels of FXR in colon cancer cell lines. (^∗^ denotes *P* < 0.05).

Previous studies have displayed that FXR loss is associated with tumor-promoting phenotypes [31]. To investigate the correlation between FXR and colon cancer development, Wilcox analysis was conducted on FXR using TCGA database to confirm the low expression of FXR in tumor tissues (Figure 1(a)). We further performed TCGA database to analyze the association between FXR expression and TNM stage in colon cancer patients. The results exhibited that the expression of FXR had no significantly correlation with distant metastasis, regional lymph nodes, and tumor grading (Supplementary Figure 1A). Besides, FXR was obviously downregulated in T3+T4 group compared to T1+T2 group (Figure 1(b)). At the same time, we also detected the mRNA and protein expression levels of FXR in clinical colon cancer adjacent tissues and cancerous tissues by qRT-PCR and Western blot, respectively. The results showed that the mRNA and protein expression levels of FXR were significantly reduced in tumor tissues (Figure 1(c)). Subsequently, the mRNA and protein expression levels of FXR in normal colon epithelial cells and colon cancer cell lines were measured by qRT-PCR and Western blot. It was shown that the mRNA and protein expression levels of FXR were prominently lower in colon cancer cell lines (HCT 116, HT-29, SW480, Caco-2, RKO, Lovo) than in normal colon epithelial cells (NCM460). Among them, the expression level of FXR was relatively high in HT-29 cells and relatively low in SW480 cells (Figures 1(d) and 1(e)). Therefore, HT-29 and SW480 were chosen for subsequent experiments. In conclusion, the expression of FXR was low in colon cancer.

### 3.2. FXR Upregulation Inhibits the Malignant Phenotypes of Colon Cancer Cells

**Figure 2:**
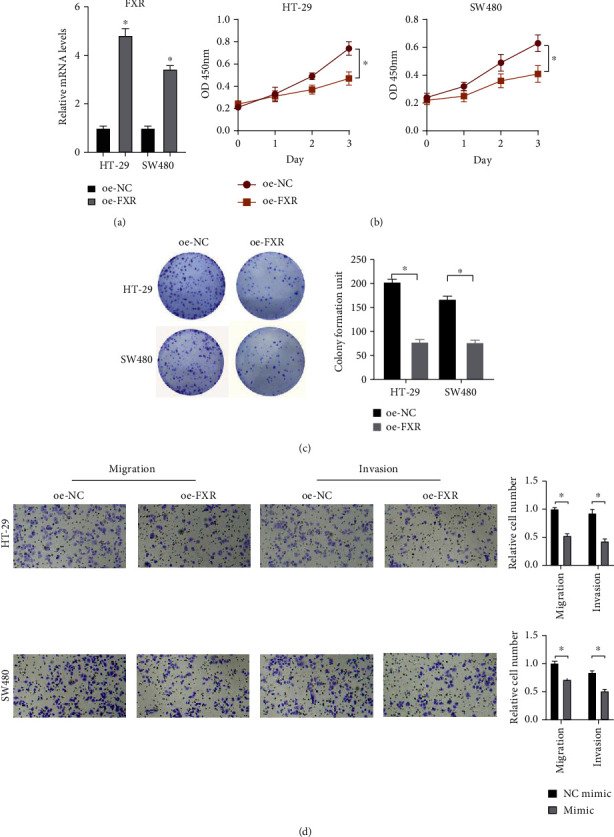
FXR overexpression inhibits malignant progression of colon cancer cells. (a) qRT-PCR confirmed the high expression of FXR in oe-FXR transfection group. (b and c) CCK-8 and colony formation assays were employed to analyze the effects of FXR overexpression on proliferation of HT-29 and SW480 cell lines. (d) The Transwell migration and invasion assays assessed the changes in motor capacity of colon cancer cells after overexpressing FXR. Each column is the average value of 3 independent experiments. (^∗^ denotes *P* < 0.05).

To verify the role of abnormal FXR expression in the biological functions of colon cancer cells, oe-NC or oe-FXR was transfected into HT-29 and SW480 cells. Transfection efficacy was evaluated by qRT-PCR, which found a remarkable increase of FXR expression in oe-FXR-transfected groups (Figure 2(a)). CCK-8 and colony formation assays were done to examine the effect of FXR on cell proliferation. Experimental data showed that FXR overexpression notably inhibited the viability (Figure 2(b)) and colony formation ability (Figure 2(c)) of HT-29 and SW480 cells compared with the control group. We then assessed the motility of oe-FXR-transfected HT-29 and SW480 cells by Transwell assays. The results demonstrated that FXR overexpression notably reduced the migratory and invasive abilities of colon cancer cells (Figure 2(d)). Taken together, FXR played a role of tumor suppressor gene and suppressed the ability of colon cancer cells to proliferate, migrate, and invade. In this work, we found that FXR had a more significant effect on the behaviors of HT-29 cells, so HT-29 cell line was used for subsequent experiments.

### 3.3. DHRS9 Is a Target of FXR in Colon Cancer

**Figure 3:**
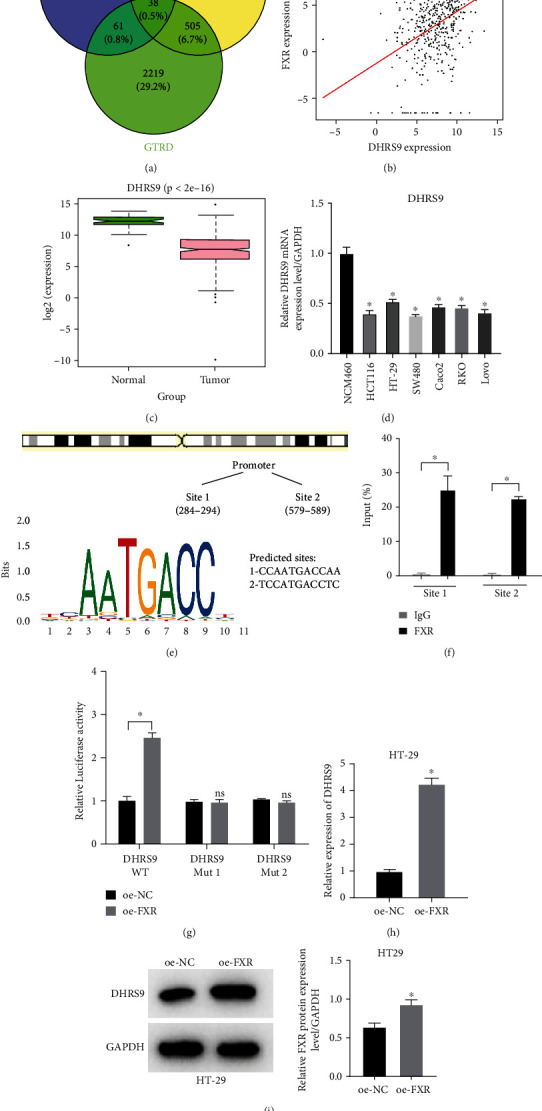
FXR activates DHRS9 and upregulates DHRS9 expression. (a) Venn diagram of FXR target genes predicted by MotifMap and GTRD databases. (b) Scatter chart of the Pearson correlation analysis between FXR and DHRS9 in TCGA database. (c) Boxplot of DHRS9 expression in normal and colon cancer tissues. (d) mRNA expression of DHRS9 in colon cancer cell lines measured by qRT-PCR. (e) Binding sequence of DHRS9 promoter region and FXR in JASPAR database. (f and g) Binding between FXR and DHRS9 promoter region demonstrated by ChIP and dual-luciferase assays. (h and i) Effect of FXR overexpression on DHRS9 expression in HT-29 cells detected by qRT-PCR and Western blot. (^∗^ denotes *P* < 0.05).

To further explore the potential mechanism of FXR, we predicted its potential downstream target genes by using MotifMap database and GTRD database and intersected the target genes with 939 downregulated DEmRNAs. According to the results, 38 differential potential target genes were found (Figure 3(a)). Subsequently, the Pearson correlation analysis was applied to detect the correlation between the 38 mRNAs and FXR (Supplementary Table 1), which showed that DHRS9 had strongest positive correlation with FXR (Figure 3(b)). Further bioinformatics analysis showed a significant reduction in DHRS9 expression in colon cancer tissues compared to normal tissues (Figure 3(c)). qRT-PCR results also showed that DHRS9 expression was remarkably lowered in colon cancer cell lines compared with normal colon epithelial cells (Figure 3(d)). JASPAR database showed that there were multiple potential binding sites for FXR on the upstream of DHRS9 transcript (Figure 3(e)), so we conjectured that DHRS9 was a potential target gene for FXR. ChIP and dual-luciferase assays were employed to verify the interaction between FXR and DHRS9. The results showed that FXR bound to the DHRS9 promoter and enhanced the luciferase activity of the vector carrying the DHRS9 promoter (Figures 3(f) and 3(g)), suggesting that DHRS9 was a direct target of FXR. Finally, and HT-29 cells were treated with overexpression FXR, and then the effects of overexpression FXR on DHRS9 were examined by qRT-PCR and Western blot. The results showed that FXR overexpression could significantly increase the levels of DHRS9 mRNA and protein in HT-29 cells (Figures 3(h) and 3(i)). Through the above assays, we confirmed that FXR could activate the transcription of DHRS9 and upregulate the expression of DHRS9.

### 3.4. FXR Activates DHRS9 to Inhibit the Malignant Progression of Colon Cancer

**Figure 4:**
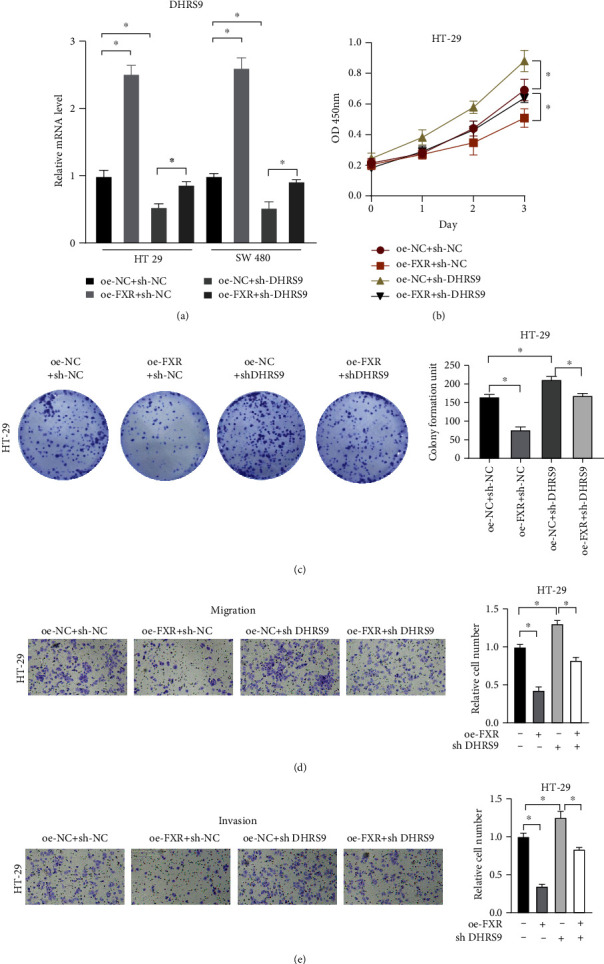
FXR activates DHRS9 to inhibit the malignant progression of colon cancer. (a) DHRS9 expression in transfected colon cancer cells detected by qRT-PCR. (b and c) Effect of FXR overexpression on HT-29 cell proliferation after DHRS9 expression blocking detected by CCK-8 and colony formation assays. (d and e) Changes in motor capacity of FXR overexpressed colon cancer cells after DHRS9 expression blocking assessed by Transwell migration and invasion assays. Each column is the average value of 3 independent experiments. (^∗^ denotes *P* < 0.05).

To investigate the role of the FXR/DHRS9 regulatory axis at the cell functional level, we designed a rescue experiment. Firstly, we transfected colon cancer cells HT-29 with oe-NC+sh-NC, oe-FXR+sh-NC, oe-NC+sh-DHRS9, and oe-FXR+sh-DHRS9. We first verified the transfection efficiency of colon cancer cells by qRT-PCR (Figure 4(a)) and found that FXR overexpression partially offsets the increase of cell proliferative ability and colony forming ability induced by DHRS9 silencing in colon cancer cells (Figure 4(b) and 4(c)). Meanwhile, cell functional experiments showed that the enhanced migratory and invasive abilities induced by sh-DHRS9 treatment in colon cancer cells were inhibited after overexpressing FXR simultaneously (Figures 4(d) and 4(e)). In conclusion, transcription factor FXR inhibited the malignant progression of colon cancer cells by activating DHRS9.

### 3.5. FXR Activated DHRS9 to Inhibit Oxidative Phosphorylation in Cells

**Figure 5:**
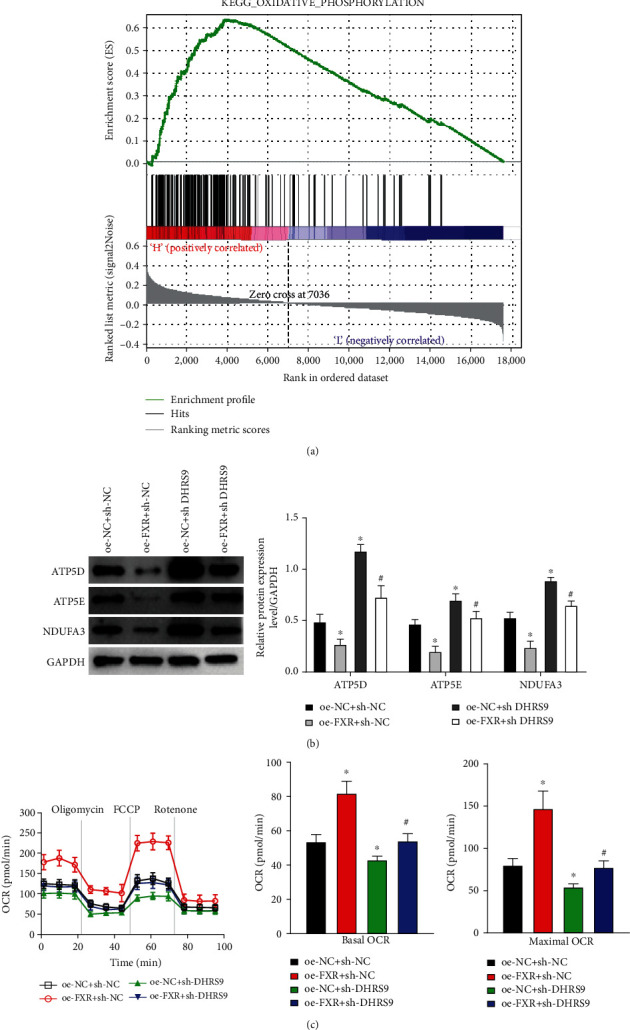
FXR activates DHRS9 to inhibit oxidative phosphorylation in colon cancer. (a) The enrichment diagram of the KEGG pathway analysis on DHRS9 gene. (b) The protein expression of oxidative phosphorylation-related genes in cells demonstrated by Western blot. (c) OCR values of different transfection groups. ^∗^ vs. oe-NC+sh-NC; # vs. oe-FXR+sh-NC (^∗^ or # denote *P* < 0.05).

We performed the KEGG pathway analysis for DHRS9 gene and found that the gene was enriched in the oxidative phosphorylation pathway (Figure 5(a)). It has been confirmed in mouse models and human clinical samples that the deficiency of mitochondrial oxidative phosphorylation changes cellular metabolism, thus accelerating the occurrence of intestinal tumors [32]. Rodríguez-Enríquez et al. [33] further found that cancer cell proliferation could be inhibited by disrupting oxidative phosphorylation and inducing oxidative stress. Based on previous studies, we first examined the protein expression of oxidative phosphorylation genes in colon cancer cells and revealed that FXR overexpression partially offset the upregulation of oxidative phosphorylation genes caused by DHRS9 expression inhibition (Figure 5(b)). Since the changes in the expression of FXR and DHRS9 in colon cancer cells led to changes in the expression of oxidative phosphorylation gene proteins, we then examined whether these changes lead to alternation in oxidative phosphorylation function. We examined OCRs of different transfected cells and found that the OCR was significantly downregulated in HT-29 cells with downregulated DHRS9 expression. Further overexpression of FXR restored the OCR level, suggesting that DHRS9 activated by FXR reduced oxidative phosphorylation-dependent OCR (Figure 5(c)). To sum up, transcription factor FXR could activate DHRS9 and inhibit oxidative phosphorylation of colon cancer cells.

## 4. Discussion

Accumulating studies have demonstrated that transcription factors are of great importance for cancer development. For example, the expression of STAT3 is dysregulated in various cancers. Inhibition of STAT3 expression in tumor cells can slow down the progression of cancer and block tumor growth and tumor cell migration [34]. KLF5 is highly expressed in basal breast cancer, and inhibiting KLF5 expression can hinder breast cancer cell migration and proliferation *in vitro* and tumorigenesis *in vivo* [35] . Herein, we found the downregulation of FXR expression in colon cancer tissues by bioinformatics analysis. As an important bile acid receptor in the nuclear receptor superfamily, FXR can interact with its ligand, the bile acid molecular, and affect the development of cancer [36] . For example, Huang et al. found that FXR inhibits the growth of HCC cells via suppressing the mTOR-s6K pathway[37] . Liu et al.[38] confirmed the role of FXR as a tumor suppressor in prostate cancer and showed that the activation or overexpression of FXR can repress the proliferation of prostate cancer cells. In the current study, we verified the low expression of FXR in colon cancer by bioinformatics analysis and molecular experiments. We further confirmed with cell functional experiments that FXR overexpression could inhibit the malignant behaviors of colon cancer cells, which agrees with the roles of FXR in other cancer studies.

Bioinformatics analysis predicted 38 potential target genes downstream of FXR. We performed the Pearson correlation analysis for 38 predicted target genes and FXR (specific data were presented in Supplementary Table 1) and found that the expression of 25 genes was positively correlated with the expression of FXR, and the expression of the other 13 genes was negatively correlated with the expression of FXR. We selected the 5 genes with the highest correlation for subsequent analysis and found that there were rare studies focused on SLC17A4/SLC51A (which ranked top 1/top 2) and colon cancer. In addition, DHRS9 has been found to be abnormally expressed in colorectal cancer [25] , but the regulatory mechanism of DHRS9 in colon cancer has not been thoroughly studied. Therefore, we selected DHRS9 as a potential target gene for our study. Studies have demonstrated that DHRS9 has a key function in colorectal cancer. Patients with low DHRS9 expression level have significantly shorter disease-free survival and significantly increased lymph node metastasis and disease recurrence[39] . DHRS9 is significantly overexpressed in pancreatic cancer tissues, and the high expression of DHRS9 is positively correlated with vascular infiltration level and associated with poor prognosis [40] . However, DHRS9 is rarely reported in colon cancer. Here, the binding between FXR and DHRS9 was confirmed by ChIP assay and dual-luciferase assay, and it was further verified that overexpression of FXR could lead to upregulation of DHRS9 expression. Cell experiments demonstrated that FXR activated DHRS9 to inhibit the malignant behaviors of colon cancer.

Oxidative phosphorylation is attracting increasing attention [41, 42] . In the current study, enrichment of the FXR/DHRS9 pathway showed that this regulatory axis was associated with oxidative phosphorylation in cells. Tan et al. [43] found that inhibiting the oxidative phosphorylation of hepatocellular carcinoma cells could reduce cell viability. Litvak et al. [32] found that the increase of oxidative phosphorylation of colon epithelial cells could lead to colonic ecological disorders. We evaluated the expression of oxidative phosphorylated-related proteins by Western blot and found that FXR overexpression could reverse the increase of protein expression caused by DHRS9 expression inhibition. Combined with the OCR measurement of cells and related rescue experiments, we finally determined that transcription factor FXR could activate DHRS9 to inhibit oxidative phosphorylation of colon cancer.

In-depth understanding of the pathogenesis of colon cancer is beneficial to the treatment of colon cancer patients. In this study, transcription factor FXR was found to regulate the malignant progression of colon cancer by activating target gene DHRS9, thereby inhibiting oxidative phosphorylation. This finding provided sufficient theoretical support for further understanding the molecular mechanism of colon cancer development. However, due to the limitations of experimental conditions, this study lacked animal experimental data. In subsequent study, our team plans to establish mouse models to further verify the role of FXR/DHRS9 in regulating colon cancer progression at animal and clinical levels.

## Figures and Tables

**Table 1:** qRT-PCR primer sequence.

Gene	Primer sequence (5′⟶3′)
FXR	F: TGCCCTGGTACAGCCTGAGT
	R: ACACAGACATTGCCCCTGGC
DHRS9	F: TTCCTTTGGCTGCTGACAGG
	R: ATTAGGAGGCCTAGCACCCA
GAPDH	F: GAACGGGAAGCTCACTGG
	R: GCCTGCTTCACCACCTTCT

## Data Availability

The data used to support the findings of this study are included within the article. The data and materials in the current study are available from the corresponding author on reasonable request.

## Supplementary Materials

Supplementary Materials Supplementary Figure 1A. The correlation about FXR with distant metastasis, regional lymph nodes, and tumor grading. ns means no significant difference.
