# Supplementary figures and images for: Transcription Factor FXR Activates DHRS9 to Inhibit the Cell Oxidative Phosphorylation and Suppress Colon Cancer Progression
Source: Anal Cell Pathol (Amst). 2022 Oct 26;2022:8275574. doi: 10.1155/2022/8275574 (PMC9629925; doi:10.1155/2022/8275574)

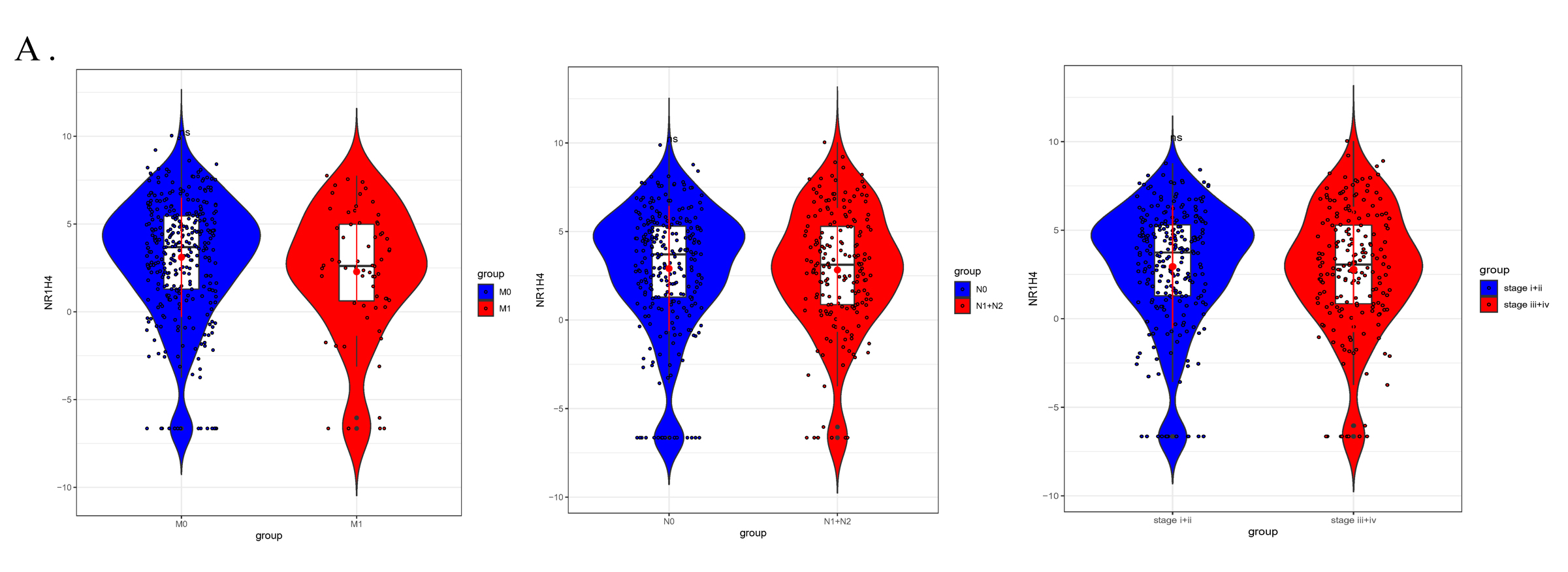

Supplement: Supplementary Materials — Supplementary Figure 1A. The correlation about FXR with distant metastasis, regional lymph nodes, and tumor grading. ns means no significant difference. [file 8275574.f1.zip › 8275574.f1.jpg]
